# Age-specific impact on the survival of gastric cancer patients with distant metastasis: an analysis of SEER database

**DOI:** 10.18632/oncotarget.21350

**Published:** 2017-09-28

**Authors:** Xinxing Li, Weijun Wang, Canping Ruan, Yi Wang, Haolu Wang, Xiaowen Liang, Yanping Sun, Zhiqian Hu

**Affiliations:** ^1^ Department of General Surgery, Changzheng Hospital, The Second Military Medical University, Shanghai, 200003, China; ^2^ Therapeutics Research Centre, School of Medicine, The University of Queensland, Princess Alexandra Hospital, Woolloongabba, QLD 4102, Australia

**Keywords:** gastric cancer, distant metastasis, age, survival

## Abstract

The age-specific impact on the survival of gastric cancer patients with distant metastasis is still unclear. In this study, we identified 11, 299 gastric cancer patients with distant metastasis between 2004 and 2013 from Surveillance, Epidemiology, and End Results population-based dataset. Patients were divided into young (≤60) and elderly groups (>60). Kaplan-Meier methods and multivariable Cox regression were used for the analysis of long-term survival outcomes and risk factors. There were significant differences between the two groups in terms of race, primary site, grade, histologic type, surgery, marital status and clinical T stage (P<0.05). The 1- and 3-year cancer specific survival rates were 29.0% and 6.2% in young group and 22.8% and 4.8% in elderly group in both univariate (X^2^=116.430, P<0.001) and multivariate analysis (P<0.001). Young patients had significantly better 1- and 3-year cancer specific survival than elderly patients in each T stage. Age was further validated as an independent survival factor in all T stages (T1, T2, T3, T4 and T_X_, P<0.05). In conclusion, age was an independent prognostic factor for gastric cancer patients with distant metastasis.

## INTRODUCTION

Gastric cancer (GC) is the fifth most common malignancy worldwide and the third leading cause of cancer deaths [1-3]. Despite the improvements in diagnosis and treatment, the 5-year overall survival (OS) rate for advanced GC is still below 30% [4-5]. Age has been recognized as an important predictor of prognosis in many cancers [6-7]. The prevalence of GC increases with age, and the peak incidence is in old population of 60-70 years [8-9]. Clarification of the relationship between age and GC survival could reveal the impact of age on cancer prognosis and improve treatment efficacy [10]. Some studies reported that young GC patients usually had advanced stage and undifferentiated tumors [11-12]. Song *et al.* [8] argued that the prognosis of GC varied with age, and young patients had a higher survival rate after surgery compared to elderly patients. Similar results were also found in Li's study [6], where young patients with colorectal cancer after surgery had a higher cancer specific survival (CSS) rate than elderly ones [6]. Although the survival and age at diagnosis in GC has been investigated [8-9, 13], age-specific impact on the survival in GC patients with distant metastasis (M1) is still unclear. In this study, we compared the pathological characteristics and prognostic outcomes of GC with M1 in young patients with elderly ones based on Surveillance, Epidemiology, and End Results (SEER) population-based data.

## RESULTS

### Patient characteristics

We identified 11, 299 GC patients with M1 diagnosed between 2004 and 2013 of the known age (≥18). In this study, we classified the patients into two groups: young (≤60) and elderly (>60) patients, including 7,128 (63.09%) males and 4,171 (36.91%) females. The average follow-up period was 5 months. Patient demographics and pathological features were summarized in Table 1.

**Table 1:** Characteristics of GC patients with M1 from SEER database

Characteristic	Total (n)	Young (≤60)	Elderly (>60)	X^2^	P value
	11299	5004	6295		
Media follow up (month)	5	6	4		
Years of diagnosis				0.003	0.955
2004-2008	5549	2456(49.1)	3093(49.1)		
2009-2013	5750	2548(50.9)	3202(50.9)		
Sex				2.953	0.086
Male	7128	3113(62.2)	4015(63.8)		
Female	4171	1891(37.8)	2280(36.2)		
Race				12.056	0.007
White	8305	3617(72.3)	4688(74.5)		
Black	1434	664(13.3)	770(12.2)		
American Indian/Alaska Native	117	66(1.3)	51(0.8)		
Asian or Pacific Islander	1443	657(13.1)	786(12.5)		
Primary site				25.720	0.001
Cardia, NOS	3552	1520(30.4)	2032(32.3)		
Fundus of stomach	463	197(3.9)	266(4.2)		
Body of stomach	1021	470(9.4)	551(8.8)		
Gastric antrum	1708	739(14.8)	969(15.4)		
Pylorus	233	85(1.7)	148(2.4)		
Lesser curvature of stomach NOS	642	269(5.4)	373(5.9)		
Greater curvature of stomach NOS	380	190(3.8)	190(3.0)		
Overlapping lesion of stomach	1061	508(10.2)	553(8.8)		
Stomach, NOS	2239	1026(20.5)	1213(19.3)		
Grade				107.261	0.000
Well/Moderately differentiated	1929	668(13.3)	1261(20.0)		
Poorly differentiated/Undifferentiated	6933	3301(66.0)	3632(57.7)		
Unknown	2437	1035(20.7)	1042(22.3)		
Histologic type				341.140	0.000
Adenocarcinoma, NOS	7338	2813(56.2)	4525(71.9)		
Carcinoma	951	441(8.8)	510(8.1)		
Signet ring cell carcinoma	3010	1750(35.0)	1260(20.0)		
Surgery				13.492	0.000
Yes	1693	819(16.4)	874(13.9)		
No	9606	4185(83.6)	5421(86.1)		
Marital status				1235.527	0.000
Married	6649	2944(58.8)	3705(58.9)		
Divorced	978	455(9.1)	523(8.3)		
Widowed	1308	90(1.8)	1218(19.3)		
Single/separated/unmarried	1927	1336(26.7)	591(9.4)		
Unknown	437	179(3.6)	258(4.1)		
T stage				44.531	0.000
T1	1814	730(14.6)	1084(17.2)		
T2	1784	809(16.2)	975(15.5)		
T3	1010	492(9.8)	518(8.2)		
T4	2316	1123(22.4)	1193(19.0)		
T_X_	4375	1850(37.0)	2525(40.1)		

### Characteristics of GC patients

There were significant differences between the two groups in terms of race, primary site, grade, histologic type, surgery, marital status and clinical T stage (P<0.05). Compared to elderly ones, young GC patients with M1 had more undifferentiated grade (66.0% VS 57.7%, P<0.05), more signet-ring cancer (35.0% VS 20%, P<0.05), and more stage T3 and T4 (9.8% VS 8.2%, 22.4% VS 19.0%; P<0.05). Except for married patients in young and elderly groups (58.8% and 58.9%), most of young ones were single/separated/unmarried (26.7%), while most of elderly were widowed (19.3%). As shown in Table 1, no significant differences were found between two groups in years of diagnosis (P=0.955) and sex (P=0.086).

### Impact of age on GC survival outcomes

**Figure 1:**
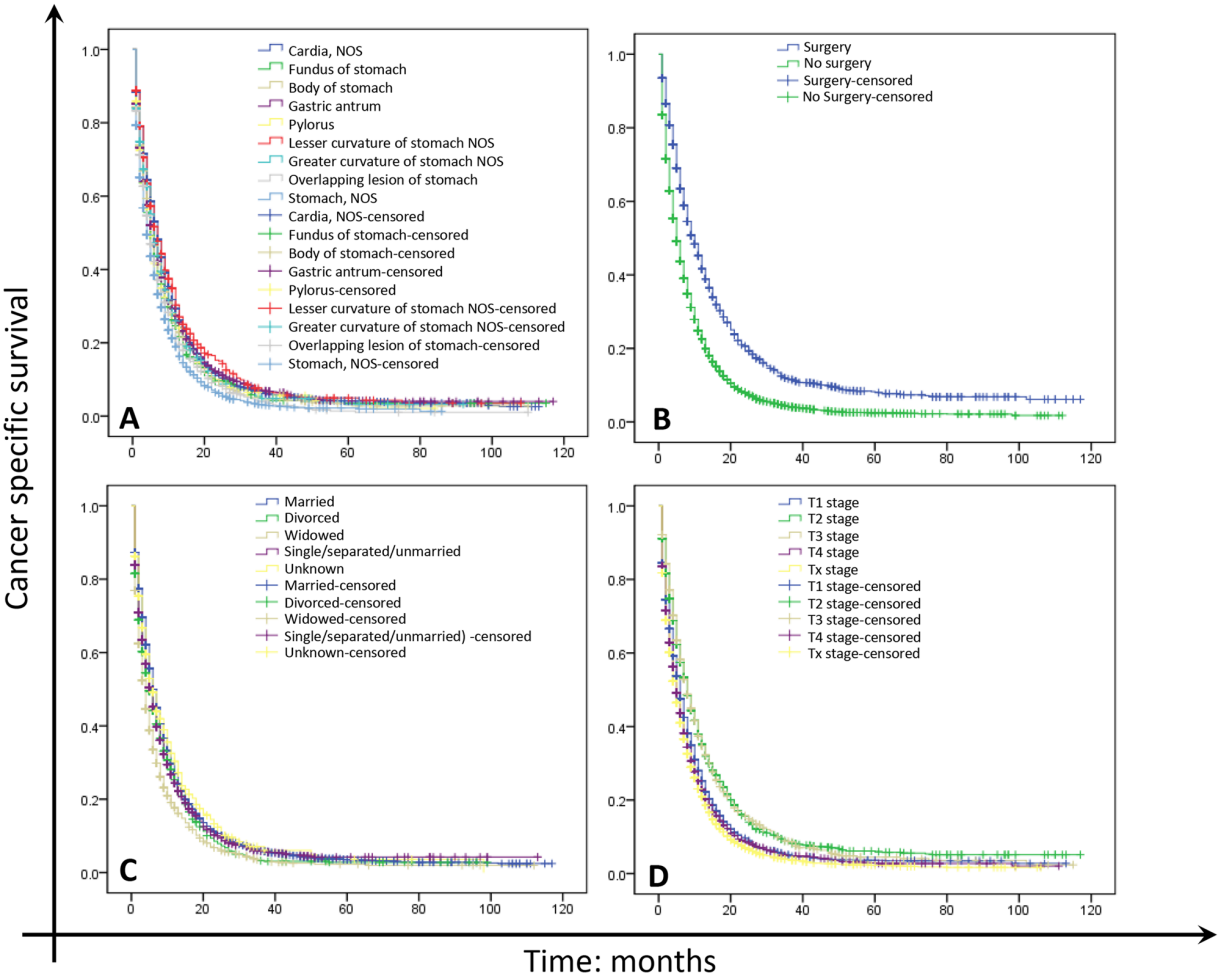
Survival curves in GC patients with M1 of different primary site, surgical treatment, marital status and T stage **(A)** Primary site. X^2^ = 162.320, P<0.001; **(B)** Surgery. X^2^ = 313.299, P<0.001; **(C)** Marital status. X^2^ = 119.561, P<0.001; **(D)** T stage. X^2^ =252.356, P<0.001.

**Figure 2:**
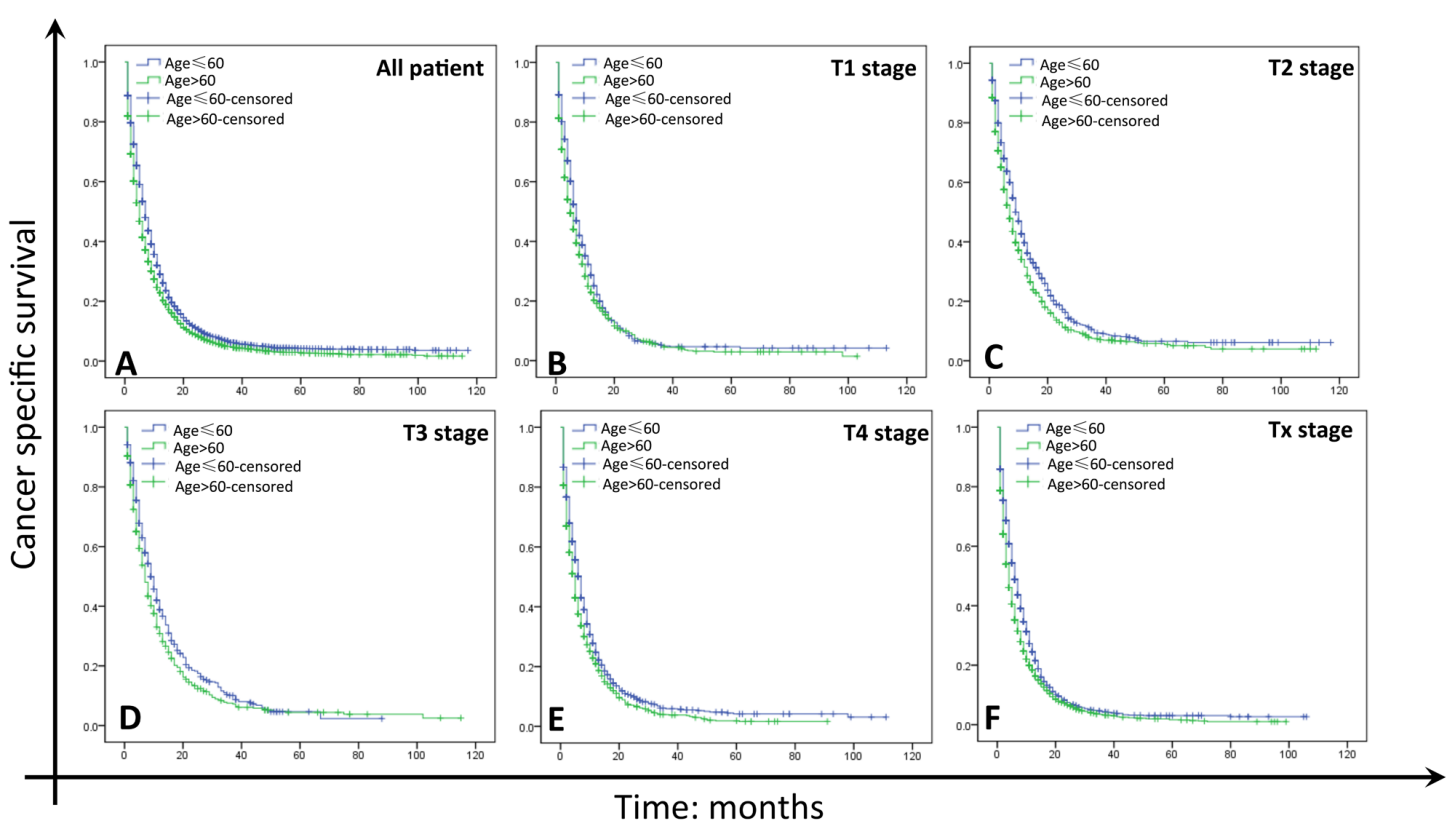
Survival curves in GC patients with M1 of different age **(A)** All patients. Young group vs. Elderly group, X^2^ = 116.430, P<0.001; **(B)** T1 stage. Young group vs. Elderly group, X^2^= 9.817, P=0.002; **(C)** T2 stage. Young group vs. Elderly group, X^2^ = 20.034, P<0.001; **(D)** T3 stage. Young group vs. Elderly group, X^2^ = 8.278, P=0.004; **(E)** T4 stage. Young group vs. Elderly group, X^2^ = 22.753, P<0.001; **(F)** Tx stage. Young group vs. Elderly group, X^2^ = 51.779, P<0.001.

As shown in Tables 2 and 3 and Figure 2, the 1- and 3-year CCS rates of GC were 29.0% and 6.2% in young group, and 22.8% and 4.8% in elderly group, which had significant difference using univariate (X^2^=116.430, P<0.001) and multivariate analysis (young group as ref., HR=0.808, 95%CI: 0.773∼0.844, P<0.001). Year of diagnosis, sex, race, primary site, grade, histological type, surgery, marital status and clinical T stage were identified as significant risk factors for poor survival by univariate analysis (Table 2 and Figure 1, P<0.05). Female, black and American Indian/Alaska Native and widowed GC patients also had shorter survival periods (Table 2 and Figure 1). As shown in Table 2, the 1- and 3-year CCS rates of patients in stage T1 (25.3% and 5.1%) were lower than that of in T2 (35.2% and 8.4%) and T3 (34.7% and 8.8%), but higher than that in T4 (22.8% and 4.9%) and Tx (21.0% and 3.8%). Multivariate analysis with Cox regression revealed that year of diagnosis, age, race, primary site, grade, histological type, surgery, marital status and pathological T stage were independent prognostic factors (Table 3, P<0.05).

**Table 2:** Univariate survival analyses of GC patients with M1

Variable	Total (n)	1-year CSS	3-year CSS	Log rank x^2^	P value
Years of diagnosis				18.913	0.000
2004-2008	5549	24%	4.8%		
2009-2013	5750	27.2%	6.3%		
Sex				5.845	0.016
Male	7128	26.3%	5.7%		
Female	4171	24.3%	4.9%		
Age				116.430	0.000
Young	5004	29.0%	6.2%		
Elderly	6295	22.8%	4.8%		
Race				19.193	0.000
White	8305	25.3%	5.2%		
Black	1434	23.1%	4.8%		
American Indian/Alaska Native	117	23.2%	4.8%		
Asian or Pacific Islander	1443	29.8%	7.2%		
Primary site				162.320	0.000
Cardia, NOS	3552	29.2%	6.5%		
Fundus of stomach	463	24.6%	4.8%		
Body of stomach	1021	25.5%	4.8%		
Gastric antrum	1708	27.4%	6.9%		
Pylorus	233	25.7%	6.4%		
Lesser curvature of stomach NOS	642	30.3%	7.0%		
Greater curvature of stomach NOS	380	26.0%	4.8%		
Overlapping lesion of stomach	1061	21.2%	4.1%		
Stomach, NOS	2239	18.9%	3.1%		
Grade				66.031	0.000
Well/Moderately differentiated	1929	32.3%	7.9%		
Poorly differentiated/Undifferentiated	6933	24.9%	4.9%		
Unknown	2437	22.0%	4.9%		
Histologic type				11.442	0.003
Adenocarcinoma, NOS	7338	26.5%	6.0%		
Carcinoma	951	23.4%	5.1%		
Signet ring cell carcinoma	3010	23.8%	4.1%		
Surgery				313.299	0.000
Yes	1693	41.6%	11.7%		
No	9606	22.6%	4.2%		
Marital status				119.561	0.000
Married	6649	27.1%	5.9%		
Divorced	978	25.3%	3.3%		
Widowed	1308	17.7%	3.3%		
Single/separated/unmarried)	1927	24.4%	5.8%		
Unknown	437	30.3%	7.0%		
T stage				252.356	0.000
T1	1814	25.3%	5.1%		
T2	1784	35.2%	8.4%		
T3	1010	34.7%	8.8%		
T4	2316	22.8%	4.9%		
T_X_	4375	21.0%	3.8%		

**Table 3:** Multivariate Cox model analyses of prognostic factors of GC patients with M1

Variable	HR	95%CI	P value
Years of diagnosis			0.000
2004-2008		Ref	
2009-2013	1.115	(1.070∼1.161)	0.000
Sex			0.900
Male		Ref	
Female	1.035	(0.989∼1.082)	0.136
Age			0.000
Young		Ref	
Elderly	0.808	(0.773∼0.844)	0.000
Race			0.008
White		Ref	
Black	1.104	(1.037∼1.175)	0.002
American Indian/Alaska Native	1.139	(1.050∼1.235)	0.002
Asian or Pacific Islander	1.281	(1.045∼1.570)	0.017
Primary site			0.000
Cardia, NOS		Ref	
Fundus of stomach	0.754	(0.709∼0.801)	0.000
Body of stomach	0.838	(0.751∼0.935)	0.002
Gastric antrum	0.842	(0.776∼0.913)	0.000
Pylorus	0.855	(0.798∼0.917)	0.000
Lesser curvature of stomach NOS	0.918	(0.792∼1.062)	0.250
Greater curvature of stomach NOS	0.750	(0.680∼0.828)	0.000
Overlapping lesion of stomach	0.854	(0.758∼0.962)	0.009
Stomach, NOS	0.950	(0.877∼1.029)	0.207
Grade			0.001
Well/Moderately differentiated		Ref	
Poorly differentiated/Undifferentiated	0.880	(0.822∼0.943)	0.000
Unknown	1.044	(0.991∼1.099)	0.103
Histologic type			0.003
Adenocarcinoma, NOS		Ref	
Carcinoma	0.945	(0.899∼0.995)	0.30
Signet ring cell carcinoma	1.003	(0.926∼1.086)	0.946
Surgery			0.000
Yes		Ref	
No	0.638	(0.597∼0.681)	0.000
Marital status			0.000
Married		Ref	
Divorced	1.076	(0.963∼1.201)	0.196
Widowed	1.234	(1.087∼1.401)	0.001
Single/separated/unmarried)	1.371	(1.211∼1.552)	0.000
Unknown	1.194	(1.061∼1.344)	0.003
T stage			0.000
T1		Ref	
T2	0.922	(0.868∼0.979)	0.008
T3	0.790	(0.740∼0.842)	0.000
T4	0.852	(0.785∼0.942)	0.000
T_X_	0.999	(0.944∼1.056)	0.962

### Stratified analysis of age on GC survival based on T stage

We then further analyzed the effect of age on 1- and 3-year CSS at each clinical T stage. We found that young patients had significantly better 1- and 3-year CSS than elderly patients in each T stage (Table 4, P<0.05). Age was further validated as an independent survival factor in multivariate Cox regression at T1 stage (elderly, HR: 0.824, 95%CI: 0.742∼0.915; P<0.001), T2 stage (elderly, HR: 0.756, 95%CI: 0.680∼0.841; P<0.001), T3 stage (elderly, HR: 0.818, 95%CI: 0.713∼0.938; P=0.004), T4 stage (elderly, HR: 0.811, 95%CI: 0.742∼0.886; P<0.001) and Tx stage (elderly, HR: 0.784, 95%CI: 0.733∼0.838; P<0.001) patients (Table 4).

**Table 4:** Univariate and multivariate analysis of age on CSS of different T stages

Variable	Total (n)	1-year CSS	3-year CSS	Univariate analysis		Multivariate analysis	
				Log rank x^2^	P value	HR (95%CI)	P value
**T1**	1814			9.817	0.002		
Age							
Young	730	28.7%	5.2%			Ref	
Elderly	1084	23.0%	5.0%			0.824(0.742∼0.915)	0.000
**T2**	1784			20.034	0.000		
Age							
Young	809	39.6%	9.4%			Ref	
Elderly	975	31.5%	7.5%			0.756(0.680∼0.841)	0.000
**T3**	1010			8.278	0.004		
Age							
Young	492	38.8%	10.1%			Ref	
Elderly	518	33.0%	7.5%			0.818(0.713∼0.938)	0.004
**T4**	2316			22.753	0.000		
Age							
Young	1123	24.7%	5.9%			Ref	
Elderly	1193	20.9%	3.9%			0.811(0.742∼0.886)	0.000
**Tx**	4375			51.779	0.000		
Age							
Young	1850	24.5%	4.3%			Ref	
Elderly	2525	18.5%	3.4%			0.784(0.733∼0.838)	0.000

P values refer to comparison between two groups and were adjusted for years of diagnosis, sex, race, primary site, grade, histological type, surgery, marital status and clinical T stage.

## DISCUSSION

Despite advancement of diagnosis and treatment of GC, the prognosis remains poor with a 5-year OS of less than 30% in most countries [4-5, 14]. In China, GC is the second leading cause of cancer death, and the current 5 year CSS is low because more than 80% of patients are diagnosed at an advanced stage [15-16]. Age is considered as one of the independent factors of several cancers [6-9, 12]. Investigation of important prognostic factors of GC development could further understand and improve the treatment of the advanced disease. We identified 11, 299 GC patients with M1 diagnosed between 2004 and 2013 with a known age based on SEER population-based data. The current definition of elderly patients remains controversial. Some studies used the cutoff age of 50 years, while others used 30 years or 45 years [6, 8-9, 17]. In this study, we divided GC patients into young (≤60) and elderly (>60) groups according to recent publications and the new age subsection-standard of the United Nations world health organization [8-9].

Recently, some studies have investigated the prognostic outcome of GC in young patients in comparison to the elderly, but yielded inconclusive results [18-19]. It has been suggested that young patients suffered worse survival due to the characteristics of themselves and different tumor behavior [20]. Chen *et al.* [9] reported that between 56 and 65 years have more favorable clinicopathologic characteristics and better CSS than the other groups in operable gastric cancer patients. While Song *et al.* [8] argued that the prognosis of GC varied with age, and young patients suffered a higher survival rate after surgery compared to elderly patients. In our study, we found that young GC patients with M1 had a higher CCS rate compared to elderly ones. The 1- and 3-year CCS rates of GC were 29.0% and 6.2% in young group and 22.8% and 4.8% in elderly group, which had significant difference by univariate (X^2^=116.430, P<0.001) and multivariate analysis (young group as ref., HR=0.808, 95%CI: 0.773∼0.844) (P<0.001). It might be attributed to two main reasons. One could be explained by that the poor tolerance of extensive lymphadenectomy and standardized chemotherapy [21-22]. Clinicians are more likely to provide all remedial options for young patients since they have the better health condition and tolerance of chemotherapy [31]. The other reason was that young patients usually have better tolerance of surgery and better recovery [23-24]. Although some ones thought that oldness would another reason for affecting long-time survival, most of GC patients with distant metastasis died in 1 year and 3-year CSS was less than 10%.

Another interesting finding was that compared to elderly ones, young GC patients with M1 had characteristics of more poor or undifferentiated grade (66.0% VS 57.7%, P<0.05), more signet-ring cancer (35.0% VS 20%, P<0.05), more stage T3 and T4 (9.8% VS 8.2%, 22.4% VS 19.0%; P<0.05). Li *et al.* [6] also found young patients presented higher proportions of unfavorable behavior as well as advanced stage disease. In contrast, it was noted that that young patients suffered worse survival due to the personal characteristics and different tumor behavior [20]. It is well known that mucinous, signet-ring and poorly differentiated tumors tend to have a poorer prognosis compared to well and moderately differentiated tumors [25]. It is thought that gastric cancer results from a combination of environmental factors and an accumulation of specific genetic alterations. The genetic information of young patients is different from that which leads to sporadic carcinomas at an older age. And there is a tendency of late diagnosis of the disease in young patients [26-27].

In addition, we found that except for married ones in young and elderly groups (58.8% and 58.9%), most of young GC patients were single/separated/unmarried (26.7%), while most of elderly were widowed (19.3%) who had the shortest survivals. Li *et al.* [28] selected 112, 776 colorectal cancer from SEER data and found unmarried patients were at greater risk of cancer specific mortality while widowed patients were at the highest risk of death than the other groups. Jin *et al.* [29] suggested that marriage had a protective effect against under-treatment and cause-specific mortality in GC. It might be attributed to that widowed patients lack of social and connubial support [30] and psychosocial distress [31]. Widowed cancer patients showed more distress, depression, and anxiety than married counterparts, which might be attributed to that spouse could share the emotional burden and provide appropriate his/her support [32]. Depression or/and nonadherence have been found to be directly correlated to widowed cancer individuals [33]. It was reported that depression was related to VEGF, stimulating endothelial cell migration, proliferation and proteolytic activity in cancers [34]. Depression was strongly influenced by poor adherence to medical treatment.

This study has several limitations. First, the SEER database does not include information of therapeutic options such as detailed information of chemotherapy, targeted therapy, immunotherapy, recurrence and metastasis, which may also impact patients’ prognosis [35]. Second, the SEER database is lack of detailed description of the organ metastasis (liver, lung, bone or brain). Third, since most patients did not receive operation, we used clinical T stage instead of pathological T, which also may affect the analysis of prognosis in this study. Despite these limitations, we first reported that age was an independent prognostic factor in GC patients with M1. Further studies are needed to verify our findings.

## MATERIALS AND METHODS

### Study population and data extracted

The SEER database and SEER-stat software (SEER^*^Stat 8.3.2) were used to search GC patients with M1 between 2004 and 2013 with a known age (≥18). Years of diagnosis, sex, race, primary site, grade, histological type, surgery, marital status, clinical T stage, and CSS were extracted from the SEER database. Histological types were limited to adenocarcinoma (8140/3), carcinoma (8010/3; 8020/3; 8021/3 and 8145/3) and signet ring cell carcinoma (8490/3). Survival time was calculated from the date of diagnosis to the date of cancer-specific death. The exclusion criterions included: age<18, no evaluation of histological type, multiple malignant neoplasms, died within 30 days or information on CSS and survival months unavailable.

### Statistical analysis

Baseline characteristics were compared using the X^2^ test for nominal variables. Survival curves were generated using Kaplan–Meier analyses, and the differences between the curves were analyzed by log-rank test. Cox regression models were built for analysis of risk factors for survival outcomes. Statistical analyses were performed using the statistical software package SPSS for Windows, version 19.0 (SPSS Inc., Chicago, IL, USA). All P values were two-sided. P<0.05 was considered statistically significant.
